# “Janus-Faced” α-Synuclein: Role in Parkinson’s Disease

**DOI:** 10.3389/fcell.2021.673395

**Published:** 2021-05-28

**Authors:** Bipul Ray, Arehally M. Mahalakshmi, Sunanda Tuladhar, Abid Bhat, Asha Srinivasan, Christophe Pellegrino, Anbarasu Kannan, Srinivasa Rao Bolla, Saravana Babu Chidambaram, Meena Kishore Sakharkar

**Affiliations:** ^1^Department of Pharmacology, JSS College of Pharmacy, JSS Academy of Higher Education & Research, Mysuru, India; ^2^Centre for Experimental Pharmacology and Toxicology, Central Animal Facility, JSS Academy of Higher Education & Research, Mysuru, India; ^3^Division of Nanoscience & Technology, Faculty of Life Sciences, JSS Academy of Higher Education & Research, Mysuru, India; ^4^Institut National de la Santé et de la Recherche Médicale, Institute of Mediterranean Neurobiology, Aix-Marseille University, Marseille, France; ^5^Department of Protein Chemistry and Technology, CSIR-Central Food Technological Research Institute, Mysuru, India; ^6^Department of Biomedical Sciences, School of Medicine, Nazarbayev University, Nur-Sultan City, Kazakhstan; ^7^Special Interest Group – Brain, Behaviour, and Cognitive Neurosciences Research, JSS Academy of Higher Education & Research, Mysuru, India; ^8^College of Pharmacy and Nutrition, University of Saskatchewan, Saskatoon, SK, Canada

**Keywords:** α-synuclein, autophagy, neurotoxicity, gut–brain axis, Parkinson’s disease

## Abstract

Parkinson’s disease (PD) is a pathological condition characterized by the aggregation and the resultant presence of intraneuronal inclusions termed Lewy bodies (LBs) and Lewy neurites which are mainly composed of fibrillar α-synuclein (α-syn) protein. Pathogenic aggregation of α-syn is identified as the major cause of LBs deposition. Several mutations in α-syn showing varied aggregation kinetics in comparison to the wild type (WT) α-syn are reported in PD (A30P, E46K, H 50Q, G51D, A53E, and A53T). Also, the cell-to-cell spread of pathological α-syn plays a significant role in PD development. Interestingly, it has also been suggested that the pathology of PD may begin in the gastrointestinal tract and spread via the vagus nerve (VN) to brain proposing the gut–brain axis of α-syn pathology in PD. Despite multiple efforts, the behavior and functions of this protein in normal and pathological states (specifically in PD) is far from understood. Furthermore, the etiological factors responsible for triggering aggregation of this protein remain elusive. This review is an attempt to collate and present latest information on α-syn in relation to its structure, biochemistry and biophysics of aggregation in PD. Current advances in therapeutic efforts toward clearing the pathogenic α-syn via autophagy/lysosomal flux are also reviewed and reported.

## Highlights

- Updated information on the structure and biochemistry of α-syn aggregation is discussed and explained with suitable examples.
- Recent evidence on mutations in α-syn (A30P, E46K, H 50Q, G51D, A53E, and A53T) and its cell-to-cell transmission and the consequent impact on PD progression is compiled and discussed in detail.
- Mechanism of transport of pathogenic α-syn from gastrointestinal tract via the vagus nerve (VN) to brain and information on various clinical trials that validate the function of gut–brain axis in Parkinson’s disease (PD) is provided.
- Mechanism of cellular clearance of pathogenic α-syn via autophagy and clinical trials focusing on autophagy facilitation are discussed.
- Various reports indicate that native α-syn has physiological functions, but the mutated and aggregated forms are neurotoxic which play critical role in PD. Hence, understanding the mechanism of its origin, aggregation and cellular clearance will provide new leads in PD drug discovery.

## Introduction

Parkinson’s disease is a heterogeneous neurological disorder with progressive loss of dopaminergic neurons in the SNpc region in the brain (Nepal et al., 2019). One of the major hallmarks of PD is the accumulation and aggregation of misfolded α-synuclein (α-syn) protein to form LBs and Lewy neurites that cause disruption of cellular homeostasis, and degeneration of neurons (Mahul-Mellier et al., 2020). α-syn is a presynaptic neuronal protein encoded by *SNCA* gene and is expressed in several regions of the brain (Polymeropoulos et al., 1997; Meade et al., 2019). The presence of α-syn was observed for the first time by Maroteaux et al. (1988) in the presynaptic nerve terminals and in the neuronal nuclei, hence it was called syn (synapse) and nuclein (nucleus) (Maroteaux et al., 1988). Later, the presence of native and pathogenic α-syn was identified in organelles such as the GA (Gosavi et al., 2002; Mori et al., 2002), endolysosomal system (Lee et al., 2004), and mitochondrial surface (Li et al., 2007; Cole et al., 2008; Parihar et al., 2008). α-syn was also found to be associated with the inner membrane of mitochondria (Devi et al., 2008; Shavali et al., 2008; Ludtmann et al., 2018; Ganjam et al., 2019), ER (Cooper et al., 2006; Hoozemans et al., 2007; Colla et al., 2012a, b) and the mitochondria-associated ER membranes (MAM) (Guardia-Laguarta et al., 2014). Recently, several reports have confirmed the involvement of gut–brain axis in PD (Ghaisas et al., 2019). Gut dysbiosis affects the ENS and the contribute for aggregation of α-syn which is then transported to brain by cell-to-cell contacts exosomes (Danzer et al., 2012; Freundt et al., 2012; Kim et al., 2019; Jang et al., 2020; Siddu et al., 2020). Alternative theories also propose on the involvement of exosomal vesicles from gut microbiome in regulating host gene expression and the in aggregation of α-syn (Danzer et al., 2012; Rokad et al., 2019).

The synuclein family consist of three protein members - α, β, and γ (Lashuel et al., 2013). α-syn and β-syn are primarily found in brain, whereas, γ-syn in the neoplastic tissues (Zhang et al., 2011).

Although the physiological function of α-syn is still not clearly understood, reports suggest that it plays a significant role in neuronal plasticity (Uversky, 2008; Lashuel et al., 2013; Wu J.-Z. et al., 2019) and in dopamine synthesis by regulating TH (Peng et al., 2005). α-syn activates protein phosphatase 2A (PP2A), a serine/threonine phosphatase, that dephosphorylates TH (Leal et al., 2002; Peng et al., 2005; Hua et al., 2015; Qu et al., 2018). Additionally, α-syn is reported to modulate the release of the neurotransmitters in association with the SVs. Overexpression of native α-syn inhibits exocytosis (Logan et al., 2017) and mutations in the *SNCA* gene that encodes native α-syn are associated with PD with autosomal dominant inheritance pattern with a relatively early onset age than sporadic PD patients (Polymeropoulos et al., 1996; Ikeuchi et al., 2008). Interestingly, α-synucleinopathies are reported increase in the propensity of many neurodegenerative diseases including multiple system atrophy (MSA), Lewy body dementia (LBD) and NBIA Type 1 (formerly known as Hallervorden-Spatz disease) (Jellinger, 2003). Higher Aβ and tau expressions are reported in cortex and striatum in dementia with Lewy bodies (DLB) compared to PD (Jellinger and Korczyn, 2018). On the other hand, the hallmark histopathology feature of MSA is accumulation of α-syn in the cytoplasm of oligodendroglial cells (Dickson, 2012; Kim et al., 2014). Also, MSA has aggregated α-syn inclusions in the nuclei, unlike PD (Lin et al., 2004). Aggregation of α-syn is often observed with hyperphosphorylated Tau, transactive response DNA binding protein 43 kDa (TDP-43), Aβ, and prion protein accumulation in brain (Visanji et al., 2019). The missense mutations in *SNCA* cause the substitutions of G51D and A53E that result in atypical synucleinopathies (mixture of PD and MSA pathologies) (Schweighauser et al., 2020). Due to the conflicting reports, the physiological and pathophysiological role of α-syn aggregation remains elusive.

Fluorescent labeling report showed the presence of α-syn in several brain regions such as OB, dorsal nucleus of the VN, amygdala, hippocampus, and neocortex, besides SNpc (Braak and Del Tredici, 2009). PD was reported to spread to the connected regions (Braak et al., 2003b). The role of α-syn misfolding in the initiation of PD is well established (Mahul-Mellier et al., 2020). Several studies report on targeting α-syn aggregation and synthesis as a potential therapeutic option in PD (Fields et al., 2019; Chakroun et al., 2020; Gabr and Peccati, 2020; Mahul-Mellier et al., 2020; Ryan et al., 2020). Here, it is important to mention that native α-syn plays a crucial role in releasing the neurotransmitter associated with SV due to its greater curvature, but its over-expression is reported to inhibit the release of neurotransmitters (Sulzer and Edwards, 2019; Cai et al., 2020). Furthermore, mitochondrial localized monomeric α-syn is reported to enhance the bioenergetics of mitochondria (Ludtmann et al., 2016), but the oligomeric form causes detrimental effects and results in mitochondrial dysfunction, particularly in PD (Tripathi and Chattopadhyay, 2019). Here, it must be noted that although misfolded α-syn is reported as a major hallmark of PD, several physiologies associated with native α-syn remain elusive. Hence, there is a need to collate information about this protein from literature and revisit its physiological functions, mechanism of aggregation and its autophagic clearance in view of the latest reports. In this review, we have attempted to summarize the functions of α-syn and various therapeutic approaches that target this protein in PD.

## α-Synuclein – Structure and Biochemistry

### Structure

**FIGURE 1:**
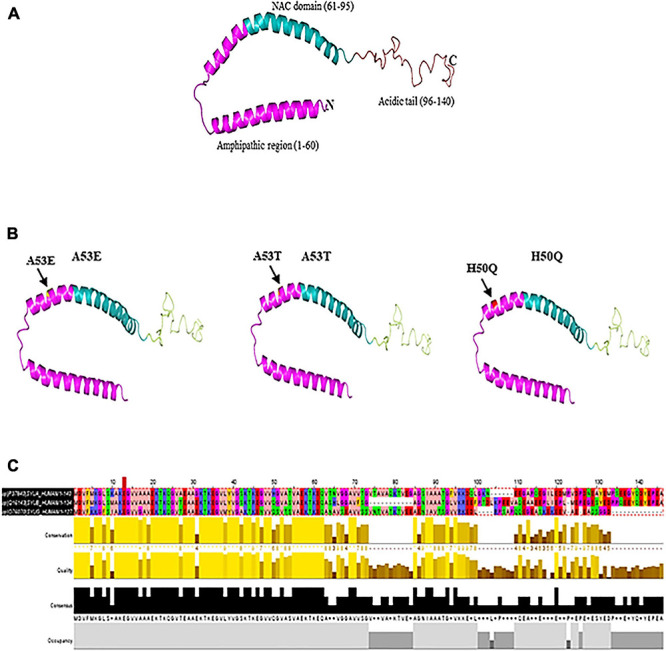
**(A)** Native α-syn structure (PDB:1XQ8). **(B)** Mutation regions (A53E, A53T, and H50Q) of α-syn. **(C)** Multiple sequence alignment of synuclein (α, β, and γ). Residues are colored according to Zappo color scheme.

The discovery of α-syn using an antibody against the purified cholinergic vesicles from the Torpedo electric organ (Torpedo Californica) gave the first evidence of its existence in the presynaptic nerve terminal (Maroteaux et al., 1988). Native α-syn is normally released α-syn into the extracellular space as exosome via exocytosis (Lee et al., 2005). It is a 14 kDa protein with 140 amino acids and three domains: a N-terminal domain (amino acid 1–60) with incomplete KXKEGV motifs, a non-amyloid-β component of plaques (NAC) domain (amino acid 61–95), and a C-terminal domain (amino acid 96–140) (pKa of 4.7) (Jakes et al., 1994; Hashimoto and Masliah, 1999; Lashuel et al., 2013). Protein containing the NAC (non-amyloid component) domain undergoes three state transition from original structure to β-sheet and further to α-helical structure (Figure 1). Other two domains undergo transformation from native to α-helical structure. Being highly hydrophobic, β-sheet reacts with 1-anilinonaphthalene-8-sulfonic acid to undergo self-aggregation (Li et al., 2002). Native α-syn exists in high concentration in soluble and membrane associated fraction in the brain and makes for as much as 1% of the total protein in the soluble brain cytosolic protein (Iwai et al., 1995). It is predominantly present in the presynaptic brain and cerebrospinal fluid (CSF) (Rokad et al., 2017). The native form of α-syn is a monomer. However, there is a possibility that the protein could form oligomers upon interaction with other proteins.

#### Oligomerization of α-Synuclein

Depending on the aggregation conditions of α-syn, heterogenous and diverse oligomers are formed, which can be identified by their biophysical and cellular properties. Danzer et al. (2007) had studied three different aggregation protocols of oligomerization of α-syn: where type A had high membrane permeability initiating an elevation of intracellular calcium (Adamczyk and Strosznajder, 2006; Lashuel and Lansbury, 2006) and leading to cell death, whereas, type B and C are able to enter cells directly and seed intracellular α-syn aggregation (Danzer et al., 2007). The report also confirmed that, type B and C do not induce caspase activation or cell loss. It is suggested that this loss might be because of bypassing the toxic oligomeric intermediates aggregation, which might have formed due to α-syn overexpression (Danzer et al., 2007). Along with the ability of self-assemble of α-syn into a variety of oligomeric species, α-syn has been further reported to interact with other proteins undergoing co-oligomerization, including Aβ and tau (Mandal et al., 2006; Qureshi and Paudel, 2011; Sengupta et al., 2015; Chia et al., 2017). In another study, β-sheet geometry between different oligomeric species was reported (Chen et al., 2015) despite mutant variants (A53T, A30P, and E46K) producing similar concentrations and types of oligomeric species to WT protein (Tosatto et al., 2015). High degree of heterogeneity of β-sheet oligomers with the same type of core architecture with different number of β-strands and arrangements and permutations of inter-strand hydrogen bonding interactions is expected as has been observed to occur in fibrillar structures (Qiang et al., 2012; Fitzpatrick et al., 2013). Indeed, the same protein subunits within the same oligomeric species might have different numbers and lengths of β-strands, in the packing of the oligomers and the rearrangement of the β-strands from an antiparallel to a parallel configuration, which might be important for the efficient elongation of these α-syn oligomers to generate the fibrillar architecture (Chen et al., 2015). Recently, Kiechle et al. (2019) had reported that, despite the availability of oligomerization of α-syn throughout the neuronal cell, the oligomerization takes place at the pre-synapse in an animal model of PD (Kiechle et al., 2019). Further in a similar study it was reported that α-syn oligomers accumulate within synaptic terminals of autonomic fibers of the skin in PD patients, which could potentially be a reliable biomarker for detecting the disease (Mazzetti et al., 2020).

### Biochemistry

Despite decades of research, the structure and functional relationship of endogenous physiological forms of native α-syn are not elucidated. Anderson et al. (2006) investigated the property of misfolded α-syn in LBs isolated from DLB brains. The phosphorylation of α-syn remains elusive, although kinases including polo-like kinase (PLK), casein kinase (CK)1, CK2, G protein coupled receptor kinase (GRK) families were identified to mediate this event (Dzamko et al., 2014). 90% of α-syn from PD brains is reported to be phosphorylated while 4% phosphorylation of α-syn is observed in normal brains (Dzamko et al., 2014). Phosphorylation of α-syn at Ser 129 has gained a significant importance in the pathogenic aggregation of α-syn (Fujiwara et al., 2002; Paleologou et al., 2008; Ma et al., 2016; Wang et al., 2019). Minor alterations in ubiquitination at Lys residues 12, 21, and 23 and specific truncations at Asp 115, Asp 119, Asn 122, Tyr 133, and Asp 135 are also seen (Anderson et al., 2006). Some studies have suggested that phosphorylation at Ser 129 triggers α-syn-mediated cellular toxicity (Chen and Feany, 2005; Sato et al., 2011; Karampetsou et al., 2017). However, conflicting reports suggest that phosphorylation at Ser 129 promotes proteasomal or autophagic clearance of aggregated α-syn (Gorbatyuk et al., 2008; Machiya et al., 2010; Kuwahara et al., 2012; Nübling et al., 2014; Arawaka et al., 2017). Hence, the exact role Ser 129 phosphorylation in synucleinopathies needs to be investigated.

α-Synuclein undergoes partial folding in the early stages of fibril formation (Uversky et al., 2001). Due to its structure, interactions, and sensitivity to the environment, α-syn is prone to misfolding. Amyloid fibrils can be formed from α-syn upon alterations in pH (Buell et al., 2014), temperature (Uversky et al., 2001), salt concentrations (Munishkina et al., 2004), air-water interference (Campioni et al., 2014), and contact with negatively charged lipid membranes (Galvagnion et al., 2015). Recently, Kurochka et al. (2021) had reported that formation of fibrils from α-syn monomers is significantly decreased in presence of lipids (Kurochka et al., 2021). Anions are also reported to induce partial folding of α-syn at neutral pH (Munishkina et al., 2004). Molecular crowding, i.e., intracellular increase in the concentration of macromolecules (proteins, nucleic acids, and carbohydrates) beyond 400 gram/liter in a cell has been reported to lead to the (Munishkina et al., 2004) production of intrinsically disordered proteins which tend to aggregate. To understand the impact of molecular crowding on α-syn aggregation, Bai et al. (2017) used Ficoll70^TM^ and Sucrose as crowding agents. However, the data did not provide sufficient evidence supporting the role of molecular crowding in α-syn aggregation in PD (Bai et al., 2017).

## α-Synuclein Aggregation, Transport and Propagation

The exact process of α-syn aggregation is not elucidated. Reports on random and instant transformation of the α-syn structure to the unfolded or partially folded state are available (Uversky, 2007). Under physiological conditions, native α-syn has a tendency to remain folded (Bartels et al., 2011; Wang et al., 2011). However, there is other report also, that says native α-syn is large unstructured monomer and they are aggregation prone (Burré et al., 2013). Further research in this filed will give the clear turn of the debate. The first evidence on the role of misfolded α-syn and amyloid β (Aβ) in neurodegenerative diseases was in the brains of AD patients (Uéda et al., 1993). The cleaved peptide from the plaques in AD brains was reported to be the central hydrophobic core of α-syn and was given the description of ‘non-amyloidogenic component,’ or NAC region (Uéda et al., 1993). Native α-syn requires the presence of interacting partner for aggregation in response to environmental stress, as this involves changes in the structural configuration of the protein (Weinreb et al., 1996; Uversky, 2002; Dyson and Wright, 2005; Villar-Piqué et al., 2016; Candelise et al., 2020).

**FIGURE 2:**
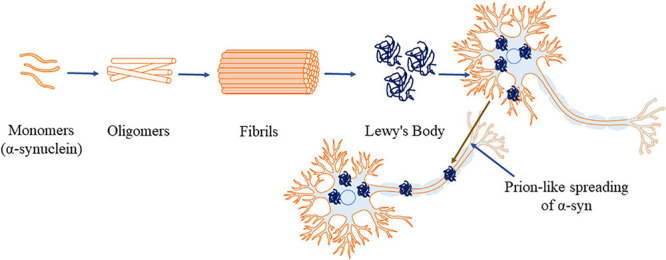
α-Synuclein aggregation process (α-syn, principal constituent of Lewy’s body). This figure was drawn using Motifolio.

Apart from interacting with other proteins, α-syn forms homo-multimers (Bartels et al., 2011; Dettmer et al., 2013, 2015). Native α-syn also self-assembles and forms β-pleated sheets (Serpell et al., 2000) which further lead to the formation of insoluble aggregations (Conway et al., 1998). NMR spectroscopy, AFM and circular dichroism have revealed the presence of increased β-sheet structure during α-syn aggregations (Pfefferkorn et al., 2012; Lashuel et al., 2013). The factors that are involved in the process of forming insoluble α-syn aggregation include genetic mutation (Conway et al., 1998; Fredenburg et al., 2007), molecular crowding induced by high concentration of macromolecules (Conway et al., 1998; Shtilerman et al., 2002; Uversky, 2002, 2007), alterations in temperature and low p^*H*^ (Ahmad et al., 2012). Additionally, minute variations in ionic strength of α-syn (25 instead of 50 mM NaCl) (Sandal et al., 2008; Roeters et al., 2017), oxidative stress (Hashimoto et al., 1999), proteins with lipid bilayer surface (Burke et al., 2013) or phospholipids (Bodner et al., 2009) also contribute to the formation of aggregates. Under these alterations in the environment, α-syn forms oligomers (Figure 2), then proto fibrils and finally the insoluble fibrils (Uversky et al., 2001; Kanaan and Manfredsson, 2012). The reaction is reported to proceed in first-order kinetics (Uversky et al., 2001; Kanaan and Manfredsson, 2012), where, in each stage the product is more stable compared to the reactants, signifying the irreversible process (Uversky and Eliezer, 2009). The hydrophobic core of various α-syn point mutations (like A30P, E46K, and A53T) reduces the α-helical content prompting the aggregation of the protein (Waxman et al., 2009; Burré et al., 2012). Srivastava et al. (2020) reported the rotenone induced alterations in the environment. Rotenone increases the hydrophobicity favoring misfolding of α-syn by reducing the lag phase and triggering aggregations (Srivastava et al., 2020).

Alerte et al. (2008) reported that the aggregation of α-syn is associated with the hyperphosphorylation of PP2A in dopaminergic neurons (Alerte et al., 2008). A small amount of intracellular α-syn translocates into the lumen of vesicles which are facilitate aggregation (Lee et al., 2005). Misfolded α-syn is removed by extracellular proteolytic enzymes or is taken up by the neighboring cells especially by microglia and astrocytes, and degraded inside lysosomes (Ahn et al., 2006; Stefanis et al., 2019). Mitochondrial and proteasomal dysfunctions triggers α-syn aggregation and further activate microglial neuroinflammation associated with PD (Ciechanover and Brundin, 2003; Lee, 2003; Zhang et al., 2005). Recent studies also propose that the aggregated α-syn gets transported like prions to other neurons (Desplats et al., 2009; Luk et al., 2012). Supporting the data, George et al. (2019) have reported microglia modulated cell to cell transfer of α-syn in PD in non-inflammatory conditions (George et al., 2019). A study has reported that lipid peroxidation by-product 4-hydroxy-2-non-enal (HNE) plays a crucial role in oligomerization and cell to cell transmission of misfolded α-syn (Bae et al., 2013).

The exact mechanism of α-syn spread is yet to be clearly understood. However, secretion of exosome like vesicles has been reported to be involved in the spread of intracellular α-syn to the extracellular space of another cell (Emmanouilidou et al., 2010; Alvarez-Erviti et al., 2011). Furthermore, exosomes are also reported to be associated the formation of α-syn oligomers which are easily uptaken by the neighboring cells (Danzer et al., 2012). Microglia was reported to uptake the α-syn via receptor mediated endocytosis (Lee et al., 2008). Along with the exosomal α-syn, free oligomeric α-syn was also reported to be up taken by neighboring cells (Danzer et al., 2012). There are several reported mechanism of cell-to-cell transfer of α-syn in PD including tunneling nanotubes, trans-synaptic junctions (Lee et al., 2005; Jao et al., 2008; Jang et al., 2010; Freundt et al., 2012; Masuda-Suzukake et al., 2014; Abounit et al., 2016; Dieriks et al., 2017). Recent studies also reported that the introduction of α-syn exosomes derived from patients with synucleinopathies to cell culture and mice model leads to propagating of α-syn aggregation (Stuendl et al., 2016; Ngolab et al., 2017). The pathological α-syn is also reported to be taken up by the surrounding microglia, which causes neuroinflammation (Chang et al., 2013; Bliederhaeuser et al., 2016) and inhibits autophagy and promotes the transmission α-syn (Xia et al., 2019). On the other hand, tunneling nanotubes (TnTs), the non-adherent actin-based cytoplasmic extensions act as a membrane bridges for intercellular transport of α-syn between two cells within a short time span of 30 sec (Gousset and Zurzolo, 2009; Abounit et al., 2016; Dieriks et al., 2017; Rostami et al., 2017). The TnTs mediated transport is not restricted to α-syn and may have a general role in transportation (Dieriks et al., 2017). The spread of α-syn over long distances via axon could possibly be involved in transferring misfolded α-syn to different regions of the brain (Jensen et al., 1999; Utton et al., 2005). Neuronal cell-to-cell transmission of α-syn fibrils is reported through axonal transport (Freundt et al., 2012). Beyond the classical exocytosis of exosome in, the transportation of α-syn from cell-to-cell (Lee et al., 2005; Danzer et al., 2012) has also been suggested to happen by trans-synaptic spreading (Danzer et al., 2011; Pan-Montojo et al., 2012; Van Den Berge et al., 2019; Mezias et al., 2020).

Several drugs inhibit cell to cell transportation of aggregated α-syn (Schofield et al., 2019; Weihofen et al., 2019; Hijaz and Volpicelli-Daley, 2020). 14-3-3θ protein was reported to inhibit the cell to cell transmission of α-syn and its toxicity by reducing the oligomerization in PD (Wang et al., 2018). Liu et al. (2021) suggest on the protective role of biocompatible antioxidant nanozyme, PtCu nanoalloys (NAs) that inhibits the prion-like spreading of α-syn in PD (Liu et al., 2021).

## Mutant α-Synuclein and PD

The *SNCA* gene is located on chromosome 4. Several mutations and polymorphisms have been observed in *SNCA* gene. Alterations in *SNCA* expression levels due to mutations have been associated to PD (Rutherford et al., 2014). The link between mutant α-syn and PD was established in an autosomal dominant form of PD with the missense mutation in the chromosome 4q21-q23 (Polymeropoulos et al., 1996). The change in a single base pair in the chromosome from guanine to adenosine (G to A transition) at the position 209 of exon number 4 in *SNCA* gene results in alteration of alanine to threonine at position 53 of the α-syn protein (A53T) (Golbe et al., 1990; Polymeropoulos et al., 1997). Furthermore, A30P (Krüger et al., 1998), E46K (Zarranz et al., 2004; Sakai et al., 2019), H50Q (Appel-Cresswell et al., 2013), G51D (Lesage et al., 2013), A53E (Pasanen et al., 2014), A53V (Yoshino et al., 2017), A18T and A29S (Hoffman-Zacharska et al., 2013) mutations are also reported. Remarkably, all the recognized mutations in the *SNCA* gene occur at the N-terminus of the protein which either disrupt the membrane binding property or result in increase in the aggregation of α-syn, thereby impairing the native α-syn functions at the pre-synaptic terminal (Conway et al., 1998; Fredenburg et al., 2007; Burré et al., 2012; Lesage et al., 2013; Fares et al., 2014). Although single base changes and small indels have been reported as the most widely studies DNA variations in PD, Copy Number Variations are also emerging as a prevalent source of genetic variations in PD (La Cognata et al., 2017). For example, Singleton et al. (2003) determined that triplication of the *SNCA* genomic locus on chromosome 4q21 is associated with PD (Singleton et al., 2003).

Majority of the identified PD mutations are located within the lipid-binding domain of α-syn suggesting that alterations in lipid binding might be associated with α-syn pathology (Pineda and Burré, 2017). SNCA variants have been shown to have differential affinity in binding to the phospholipid membranes. SNCA WT and A53T were reported to bind to rat brain vesicles whereas A30P was reported not to bind to phospholipid membranes. It was proposed that mutant α-syn potentially accumulates in the cells and assembles into Lewy body filaments (Jensen et al., 1998). Later on, it was reported that familial mutant A30P had a lesser affinity and A53T had no affinity to bind lipid membranes (Perrin et al., 2000). However, subsequently, it was confirmed that A30P, but not A53T shows decreased lipid binding affinity (Bussell and Eliezer, 2004). This was suggested to be due to the disruption of local helix formation as a result of A30P mutation (Fares et al., 2014; Ysselstein et al., 2015). In other reports, A53T mutant has been shown to have reduced (Samuel et al., 2016; Robotta et al., 2017) or similar (Middleton and Rhoades, 2010) binding affinities when compared to WT α-syn. Mutations specifically in G51D (Fares et al., 2014) and A53E (Ghosh et al., 2014) have reduced phospholipid binding. E46K variant of α-syn, binds more efficiently to anionic phospholipids, while the A30P variant shows less binding, suggesting the alterations in lipid membrane binding in PD for this variant (Stöckl et al., 2008). However, H50Q mutation does not alter lipid binding affinity (Ruf et al., 2019). These observations suggest that lipid-induced generation of fibrils is highly sensitive to the specific sequence of the SNCA protein, in particular, the region encompassing residues 46–51 (Flagmeier et al., 2016). However, the question on whether α-syn aggregation occurs in lipid bound or unbound state is under investigation (Narayanan and Scarlata, 2001; Cole et al., 2002; Lee et al., 2002a; Zhu and Fink, 2003; Burré et al., 2015; Perni et al., 2017; Mori et al., 2019).

The E46K, G51D, and the H50Q mutants of α-syn protein have significantly delayed degradation compared to WT α-syn, concurring to the data on higher resistance to degradation of these mutants in fly model of PD (Mohite et al., 2018; Sakai et al., 2019). However, H50Q variant of α-syn does not affect the structure or subcellular localization of α-syn (Khalaf et al., 2014). α-syn overexpressing SH-SY5Y cells show increased toxicity and are resistant to degradation and these aggregates are enriched in A53T α-syn (Paleologou et al., 2008; Sugeno et al., 2008; Karampetsou et al., 2017). Here it is important to mention that α-syn mutant A30P has also been reported to have slower degradation rate compared to WT α-syn (Bennett et al., 1999; Kasai et al., 2008). Unlike WT α-syn, A53T mutant has tendency for early-stage aggregation by acquiring β-sheet structure (Bertoncini et al., 2005; Camilloni and Vendruscolo, 2013) during protofibril growth, explaining the early onset of familial PD (Kang et al., 2011; de Oliveira and Silva, 2019). E46K mutations also show the propensity to acquiring β-sheet structure. However, increased N-terminal and C-terminal contacts with proteins (Rospigliosi et al., 2009; Wise-Scira et al., 2013) result in more complex and compact structure compared to WT α-syn (Fredenburg et al., 2007; Wise-Scira et al., 2013; Boyer et al., 2020). In a systematic analysis, A30P mutant was shown to have reduced tendency to form inclusions in comparison to E46K and G51D mutants. This is probably due to long-range contacts between the N and C-termini that shield the central domain, which is reported to promote aggregation (Lázaro et al., 2014). Further investigations will help to understand the role of point mutations in the pathogenic aggregation of α-syn.

## Post Translational Modifications (PTMs) of α-Syn

α-Synuclein undergoes various post-translational modifications (PTMs) and plays a crucial role in PD pathology. Until now, acetylation, phosphorylation, and nitration are the key PTMs. Phosphorylation and ubiquitination have emerged as consistent markers of α-syn pathology. Apart from Ser 129, phosphorylation at Ser 87 (Paleologou et al., 2010) is also reported in α-syn aggregation. Despite strong evidences of phosphorylation, synucleinopathic lesions also contain monoubiquitinated α-syn (Tofaris et al., 2011). However, the ubiquitination mechanism needs to be understood clearly. A small proportion of aggregated α-syn is ubiquitinated, despite the presence of ubiquitin chains in LBs inclusions (Hasegawa et al., 2002). Supporting the data, transgenic mice expressing a form of α-syn unable to undergo developmentally down-regulated gene 4 (Nedd4) associated ubiquitination showed increased α-synuclein aggregation and un-ubiquitinated synucleinopathy lesions (Periquet et al., 2007).

Nitrative stress plays a critical role in α-syn aggregation. α-syn has four tyrosine residues Y39, Y125, Y133, and Y136 which are susceptible to nitration (Chavarría and Souza, 2013; Barrett and Timothy Greenamyre, 2015). Nitration of α-syn is reported as a biomarker that is the indicative of nitrative damage in the PD patients in human and animal models (Good et al., 1998; Giasson et al., 2000; Sathiya et al., 2013; Ma et al., 2019a). Stone et al. (2012) reported that overexpression of NO synthase and NO levels triggers nitration of α-syn followed by its oligomerization in neurons (Stone et al., 2012).

**FIGURE 3:**
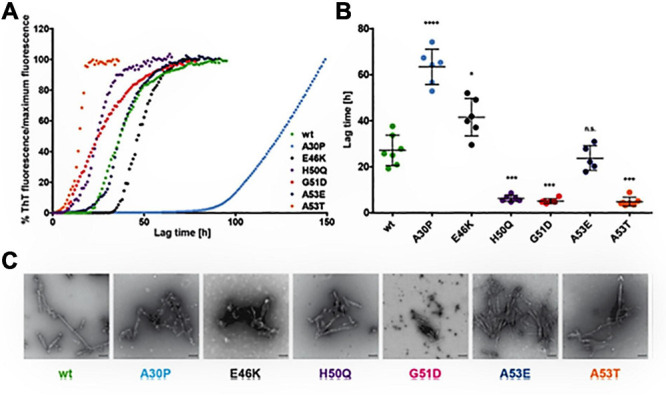
Effect of α-syn mutants on the kinetics of fibril development. **(A)** Curves indicate the aggregation kinetics of mutant α-syn, observed by ThT fluorescence over time up to 150 h. **(B)** Denotes the quantification of the lag times (time taken to show ThT fluorescence from the reference line) represent the slow fibril development of A30P and rapid fibrillation of H50Q, G51D, and A53T. E46K α-syn shows minor increase of lag times and of A53E α-syn shows no variance in fibrillation in comparison to WTα-syn. The Single dot indicates independent value (*n* ≥ 5) and the Error bars specify standard deviation, *p < 0.05, **p < 0.01, ***p < 0.001, and ****p < 0.0001. **(C)** The electron microscopy images representing fibril development of all α-syn mutants after incubation of 50 μM monomeric α-syn for 96 h at 37°C under 1400 rpm (scale bar: 200 nm). Reprinted (adapted) with permission from Ruf et al. (2019). Copyright (2019) American Chemical Society.

Oxidation of the four methionine residues: N-terminal (M1 and 5) and the C-terminal (M116 and 127) of α-syn produce methionine sulfoxides which inhibit fibrillization (Hokenson et al., 2004). Oxidative modifications of the tyrosine’s via nitration leads to the partial folded conformation that stabilizes soluble oligomers and stops elongation into fibrils (Uversky et al., 2002b, 2005; Yamin et al., 2003). In presence of H_2_O_2_ 4 methionines converts to sulfoxides (Glaser et al., 2005) and rotenone leads to methionine oxidation and subsequent intracellular aggregation (Sanders and Greenamyre, 2013).α-syn forms either antiparallel α-helices or one contiguous α-helix when interacting with acidic lipid membrane. α-syn is believed to be present as an unfolded protein in native form, which undergoes conformational change (Weinreb et al., 1996) when interacting with other molecular partners (Uversky, 2002; Dyson and Wright, 2005). Upon interaction with acidic lipid or with high curvature membrane, the N-terminus of α-syn folds into an α-helix that interacts with membranes for physiological functions (Benskey et al., 2016). Studies have reported that α-syn directly interacts with SV, SNARE complex proteins, proteins involved in calcium regulation, and the catalytic subunit of PP2A (Burré et al., 2010, 2012). Parallelly, Ruf et al. (2019) have investigated the potential of various mutant α-syn to form fibrils from monomeric α-syn (Figure 3) (Ruf et al., 2019). In conditions like genetic mutation, increased α-syn protein concentrations, post translational modifications and oxidative stress promote α-syn aggregation (Benskey et al., 2016). α-syn fibrogenesis impairs mitochondria, disrupts synapses and is toxic to the lysosome-autophagy axis and results in neurodegeneration (Lashuel et al., 2013).

It is widely acknowledged that native α-syn exists as an intrinsically disordered monomeric protein (Conway et al., 1998). However, physiologically, α-syn exists as a steady tetramer with rich α-helical structure which is immune to aggregation (Bartels et al., 2011). α-syn contains a highly amyloidogenic hydrophobic domain in the N-terminus region (amino acid 61–95), that is partly absent in β-syn (Hashimoto et al., 2001; Uversky et al., 2002a). α-syn oligomerization occurs with hydrophobic residue of the amphipathic helices to form tetrameric structures (Zhu et al., 2003; Ullman et al., 2011). Anderson et al. (2006) isolated insoluble α-syn from synucleinopathy patients to investigate changes in its primary structure in a diseased state. Adding to this data, Wang et al. (2011) reported that α-syn produced in *Escherichia coli* exists as a stable form in absence of lipids or micelles (Wang et al., 2011). However, the factors that are responsible for promoting and/or inhibiting the pathogenic α-syn accumulation are not clearly understood.

*O*-GlcNAcylation is a dynamic biochemical process, in which *N*-acetylglucosamine (GlcNAc) from uridine 5′-diphospho-*N*-acetylglucosamine (UDP-GlcNAc) is transferred to the serine and threonine residues of proteins by *O*-GlcNAc transferase (OGT) and removed by *O*-GlcNAcase (OGA) (Hart et al., 2007). *O*-GlcNAcylation identifies threonine (T) residues of α-syn isolated from mouse and human samples (Wang et al., 2010; Alfaro et al., 2012; Morris et al., 2015). *O*-GlcNAcylation at T72 completely blocks the formation of both fiber and oligomer aggregates *in vitro* (Marotta et al., 2015). The full-length α-syn with *O*-GlcNAcylation at Ser 87, aggregates with slower kinetics than the unmodified protein (Lewis et al., 2017). Several *O*-GlcNAcylated sites inhibit the toxicity of extracellular α-syn fibers that are the likely culprits in the spread of PD (Levine et al., 2019).

## α-Synuclein Pathology and Cellular Organelles in PD

It is known that α-syn aggregation is linked to various pathological cascades such as down regulation of mitochondrial complex I activity, ER stress, neuro-inflammation, disrupted cell membrane integrity, inhibition of ubiquitin proteasome system (UPS) and impaired autophagy-lysosomal pathway (ALP) in PD (Lin et al., 2019; Mahul-Mellier et al., 2020). Also, mitochondrial dysfunction plays a crucial role in the PD pathogenesis (Park et al., 2018). Interestingly, α-syn has a high affinity for mitochondrial membrane compared to other organelles (Nakamura et al., 2008; Kamp et al., 2010). Colocalization of α-syn in the mitochondrial and cytosolic fraction of rat brain tissues (Li et al., 2007) and in the SNpc and the striatum of PD patients brain is well established (Devi et al., 2008). α-syn is shown to be present in the inner mitochondrial membrane (IMM), outer mitochondrial membrane (OMM) and mitochondrial matrix (Cole et al., 2008; Liu et al., 2009; Kamp et al., 2010; Robotta et al., 2014). Its translocation to mitochondrial matrix causes alterations in complex I and increases the oxidative stress (Martínez et al., 2018). Taken altogether these data indicate that α-syn is of importance in the mitochondrial function in PD. Nevertheless, the causal link between secondary effect of PD and the associated pathogenesis is not clearly understood and needs investigation. ER is responsible for protein synthesis, folding, lipid synthesis and trafficking to Golgi. ER activates UPR when degradation of misfolded proteins is required (Walter and Ron, 2011). Proteins which fail to fold properly are degraded by proteasomes (Colla, 2019). Aggregation of α-syn triggers UPR which causes cell death (Cooper et al., 2006; Sugeno et al., 2008). Glucose regulated protein 78 (GRP78)/BiP, is the key mediator of the UPR and also senses of ER stress. Oligomeric α-syn is reported to accumulate in ER thereby triggering PD (Colla et al., 2012b; Liu et al., 2018a; Yan et al., 2019). Furthermore, the UPR activation caused by ER stress was also reported in the histopathological studies of brains of PD patients (Conn et al., 2004; Hoozemans et al., 2007; Colla, 2019).

Recently, lysosomal dysfunction, oxidative stress, and apoptosis were reported to trigger the nuclear translocations of α-syn (Ryu et al., 2019). Accumulation of α-syn in nucleus is shown to interfere with cell cycle process in PC12 cells and cause PD like motor symptoms in C57 mice (Ma et al., 2014). Mutations in *GBA* gene, which encodes for lysosomal enzyme glucocerebrosidase (GCase) is also associated with PD (Tayebi et al., 2003; Lwin et al., 2004; Gegg et al., 2020). Mutations such as N370S and L444P in GBA protein are reported in various PD patient based clinical studies (Toft et al., 2006; Marco et al., 2008; Mata et al., 2008; Hu et al., 2010; Emelyanov et al., 2012) as well as in *in vitro* (Maor et al., 2019) and *in vivo* (Taguchi et al., 2017; Yun et al., 2018) models of PD. The endo-lysosomal system regulates vesicle traffic and comprises a unique environment for proteolysis. Mutations in the endo-lysosomal protein *ATP13A2* are reported to increase the aggregation of α-syn (Lopes da Fonseca et al., 2016). Recently, Tsunemi et al. (2020) reported the impaired astrocyte mediated α-syn clearance due to the mutation in *ATP13A2* gene (Tsunemi et al., 2020). Overexpression of α-syn inhibits Ras-related protein Rab-1A (RAb1A), a GTPase, which in turn causes mis-localization of Atg9 in the TGN, an important process in autophagosome formation (Winslow et al., 2010). Mutant (A30P) α-syn suppresses c-Jun N-terminal kinase activity and inhibits autophagy in dopaminergic neurons which further increases the intracellular burden of α-syn accumulation in PD (Lei et al., 2019).

## Gut–Brain Axis and Synucleinopathy: Does PD Starts From Gut?

**FIGURE 4:**
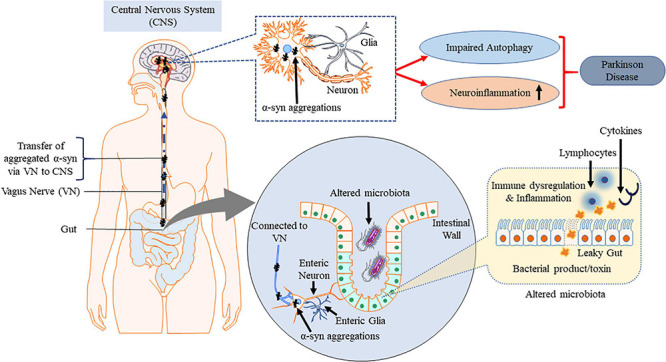
Misfolded α-syn and gut–brain axis. Gut dysbiosis, cytokines and endotoxins potentially cause inflammation at ENS which spreads to CNS via VN along with the aggregated α-syn triggered by ENS, contributing to PD pathogenesis. This figure was drawn using Motifolio.

Enteric nervous system and parasympathetic nerves get affected due to α-synucleinopathies (Edwards et al., 1992; O’Donovan et al., 2020). The VN is reported to be involved in spreading the neurogenerative process to the lower brainstem and the dopaminergic nigrostriatal system (Braak and Del Tredici, 2017; Kujawska and Jodynis-Liebert, 2018). Constipation is a common non-motor symptoms observed in the early onset of PD (Yu et al., 2018). GI dysfunction, in particular constipation, affects up to 80% of PD patients (Poewe, 2008; Cersosimo and Benarroch, 2012; Noyce et al., 2012; Müller et al., 2013). Dental deterioration, gastroparesis, delayed intestinal transit time and constipation are other symptoms associated with ENS neurodegenerative diseases (Pfeiffer, 2011; Cersosimo and Benarroch, 2012). These symptoms may appear even before the loss in motor functions and become established as early diagnostic information on PD (Braak et al., 2006; Shannon et al., 2012). The intestinal environmental factors such as the gut microbiota and the metabolites also exert their influences primarily via the gut in PD (Braak et al., 2006; Kieburtz and Wunderle, 2013; Vascellari et al., 2020). Intestinal microbiota interacts with CNS including ENS and vagal nerve (Carabotti et al., 2015; Ma et al., 2019b). Pyrosequencing of the V1–V3 regions of the bacterial 16S ribosomal RNA gene from the fecal microbiome of PD patients suggested that there are alterations in intestinal microbiome (Scheperjans et al., 2015). Extreme stimulus of innate immunity by gut dysbiosis and/or intestinal pathobionts overgrowth and the consequent increase in intestinal penetrability triggers systemic inflammation (Figure 4). Simultaneously, enteric neurons and enteric glial cells activation contribute to the aggregation of α-syn pathology (Holmqvist et al., 2014; Sampson et al., 2016). Accumulation of α-syn in PNS is reported to be associated with impairment of enteric neurons which in turn is linked to GI dysfunctions (Gold et al., 2013; Sánchez-Ferro et al., 2015). Impaired intestinal barrier integrity in PD patients increases the susceptibility of patients to microbial infections (Forsyth et al., 2011). Increased intestinal accumulation of α-syn is referred to as “leaky gut” and is common in PD patients (Chiang and Lin, 2019). Leaky gut promotes translocation of bacteria and endotoxins (bacterial products) from the gut to the brain triggering pro-inflammatory conditions and oxidative stress in the ENS (Forsythe et al., 2014). Pro-inflammatory factors associated with chronic GI disease lead to PNS inflammation which is one of the major risk factors for the observed neuroinflammation in PD (Dobbs et al., 1999; Villarán et al., 2010).

The gut bacteria synthesize many neurotransmitters as well as neuromodulators such as γ-aminobutyric acid, serotonin, dopamine, short-chain fatty acids, etc. (Lyte, 2014; Mayer et al., 2015). The gut upholds a neuronal connection via VN, as bacteria can trigger afferent neurons of the ENS (Forsythe et al., 2014). The local reflexes (migrating motor complex and peristaltic reflex) are managed by ENS via IPANs (Nezami and Srinivasan, 2010). Enteric dopaminergic neurons are present in GI and inhibit intestinal motility (Anlauf et al., 2003). The spinal cord with DMVN accepts and gives rise to the afferent and efferent fibers of the VN and influences the GI tract (Chang et al., 2003).

**FIGURE 5:**
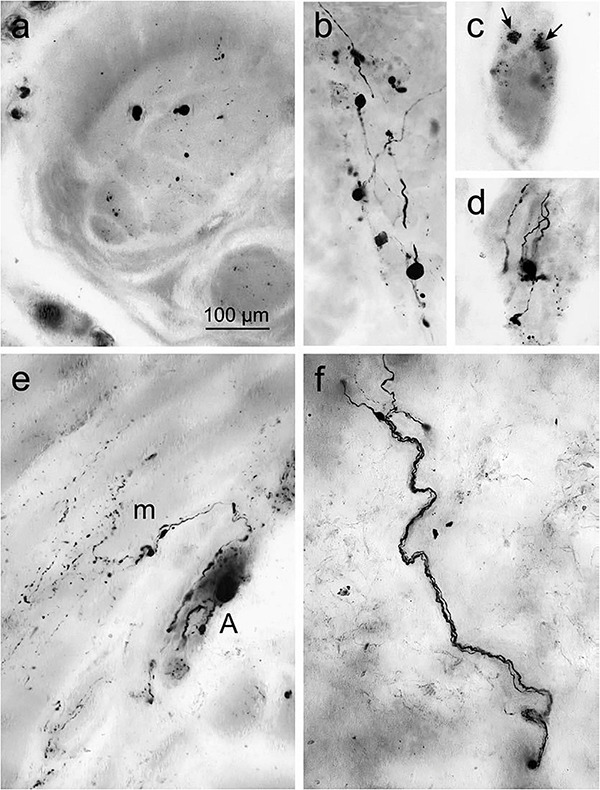
Accumulated α-syn in the surface of gastric wall. **(a)** Denotes immunoreactive inclusions in axons within the peripheral nerve. **(b–d)** Lewy neurites and LBs in the Auerbach plexus. **(b)** Presence of Lewy body pathology. **(c)** Punctate α-syn aggregations (arrows) in ENS neurons in the fundus, indicates the early signs of LBs. **(d)** Fiber-like Lewy neurites associated with the ganglia of the Auerbach plexus. **(e)** Immunoreactive fibers produced from the Auerbach plexus (A) bifurcate recurrently and fragmented into terminal ramifications along with the smooth muscle cells next to muscle layer (m). **(f)** The nerve fiber bundle of Meissner’s plexus coursing via the gastric submucosa. Reused (adapted) with the permission from Copyright Clearance Centre (License Number 5057740321467) (Braak et al., 2006).

Electrogastrography examination of patients in early and advanced PD state confirmed the persistent gastric motility irregularities (Soykan et al., 1999). Reduced amplitude of stomach contractions in PD is reported in real-time magnetic resonance imaging (Ajaj et al., 2004). The lesions in the medullar, spinal and peripheral autonomic nervous system in PD are the reasons for GI disturbances (Wakabayashi and Takahashi, 1997; Benarroch et al., 2005). In normal physiological circumstances, native α-syn is highly expressed in the CNS and is associated with regulating neurotransmission. The α-syn pathology begins in submucosal plexus of the ENS and spreads retrogradely to the CNS through vagal preganglionic axons of the DMVN (Figure 5) (Braak et al., 2006). From the DMVN a predictable caudo-rostral spread of α-syn associated pathology to other parts of the brain α-syn associated (SNpc, basal forebrain and finally neocortex region (Del Tredici et al., 2002; Braak et al., 2003b; Hawkes et al., 2007; Reichmann, 2011). This α-syn pathology spread has recently also been observed in non-human primates (Arotcarena et al., 2020). Recently, Musgrove et al. (2019) reported that oxidative stress increases at VN increasing cell to cell transmission of α-syn and promotes PD (Musgrove et al., 2019).

In early PD, phosphorylated and aggregated α-syn is identified in the ENS neurons and OB (Braak et al., 2006; Shannon et al., 2011). α-syn deposition in the neurons might start from ENS and OBs, through VN and olfactory tract, respectively (Braak et al., 2003a; Hawkes et al., 2009, 2010; Klingelhoefer and Reichmann, 2015). Interestingly, the evidence for α-syn pathology spread from the GI tract to the brain in a rat model is available (Holmqvist et al., 2014; Kim et al., 2019). Interestingly, decreased gastric motility observed in 6-hydroxydopamine-model of PD lesion in rats is also reported (Zheng et al., 2014). Also, decrease in the levels of short chain fatty acids (SCFA), the prime metabolic product of certain gut bacteria, causes alterations in the ENS and contributes to GI dysmotility in the PD (Unger et al., 2016). Lai et al. (2018) reported that a chronic low-dose MPTP may be used to assess the development of intestinal pathology as well as gut microbiota dysbiosis. This may provide new insights into the pathogenesis of PD (Lai et al., 2018; Kim et al., 2019). Many chemical signals from the gut to specific regions of the brain are also speculated to affect blood brain barrier integrity through formation of endothelial clusters, which is often recorded in PD (Guan et al., 2013). Further investigations are warranted to elucidate the exact role of the gut–brain axis in PD (Table 1).

**TABLE 1:** Clinical trials on gut–brain axis in PD.

**Study**	**Drugs**	**Phase**	**Status**	**NCT number**
Investigate the digestive microbiota and bacterial translocation during IBD and PD	–	Not applicable	Recruiting	NCT04159727
Treatment is able to restore the gut microbiota	Rifaximin	Phase 2	Recruiting	NCT03958708
Resistant maltodextrin for gut microbiome in Parkinson’s disease	Maltodextrin	Phase 2	Recruiting	NCT03667404
Brain–gut–microbiota axis and initiation of α-syn misfolding	–	Not applicable	Not yet recruiting	NCT03710668
Gut-derived neuropeptides in cerebrospinal fluid of patients with Parkinson’s disease and healthy controls	Certain gut-derived peptides (ghrelin, GLP-1)	–	Completed	NCT01792193
Effects of resistant starch on bowel habits, fecal short chain fatty acids and gut microbiota in Parkinson disease (RESISTA-PD)	Resistant starch	–	Completed	NCT02784145
Study of the genome, gut metagenome and diet of patients with incident Parkinson’s disease (16S rRNA gene sequencing)	–		Recruiting	NCT04119596
Increased gut permeability to lipopolysaccharides (LPS) in Parkinson’s disease	–	–	Completed	NCT01155492
Possible role of bacteria of the nose and gut in the pathogenesis of PD	–	–	Completed	NCT01536769
Role of gut flora in Parkinson’s disease	–	–	Recruiting	NCT04148326
Gut microbiota across early stages of synucleinopathy	–	–	Recruiting	NCT03645226
Parkinson’s disease and digestive health (neuroenteric dysfunction)	–		Recruiting	NCT04032262
Probiotics-prebiotic fiber therapy in Parkinson’s disease patients with constipation	Probiotics with prebiotic	Phase 3	Completed	NCT04451096
Study of the fecal microbiome in patients with Parkinson’s disease	PRIM-DJ2727	Phase 1	Recruiting	NCT03671785
Dietary intervention and gastrointestinal function in patients with Parkinson’s disease (MED)	Mediterranean diet	Not applicable	Active, not recruiting	NCT03851861
Fecal microbiota transplantation for Parkinson’s disease	–	–	Recruiting	NCT03808389
Study of the enteric nervous system using colonic biopsies in Parkinson patients with LRRK2 mutation (EnteroLarc)	–	–	Terminated (lack of patients)	NCT01618383

## Mechanism of Neuronal Clearance of Misfolded α-Synuclein by Autophagy

Cellular aggregation and impaired clearance of α-syn are the major pathological hallmarks of PD. Cellular clearance of misfolded proteins including α-syn is regulated by the ALP and the UPS (Lopes da Fonseca et al., 2015). Monomeric α-syn is degraded by both ALP and UPS (Liu et al., 2003; Cuervo et al., 2004) by compensatory mechanisms, i.e., when one fails the other will execute (Yang et al., 2013).

In an compensatory mechanism of ALP, a peptide based therapy protects α-syn neurotoxicity by activating proteasome pathway (Betarbet et al., 2006; Qu et al., 2020). Purified human 20S proteasomes are also reported to degrade accumulated α-syn in an ubiquitin-independent manner in PD (Tofaris et al., 2001; McKinnon et al., 2020). Supporting the data further, activation of UPS by natural alkaloid (Cai et al., 2019) and Orobol derivatives (ethanolic extracts of *Cudrania tricuspidata* fruits) is reported to decrease α-syn accumulation in PD. Additionally, phosphorylated α-syn (Ser 129) aggregates are reported to degrade via proteasome pathway (Machiya et al., 2010). Interestingly, α-syn oligomers and fibril are reported to inhibit the activity of 20S/26S proteasome subunits (Snyder et al., 2003; Zhang et al., 2008; McKinnon et al., 2020; Suzuki et al., 2020).

**FIGURE 6:**
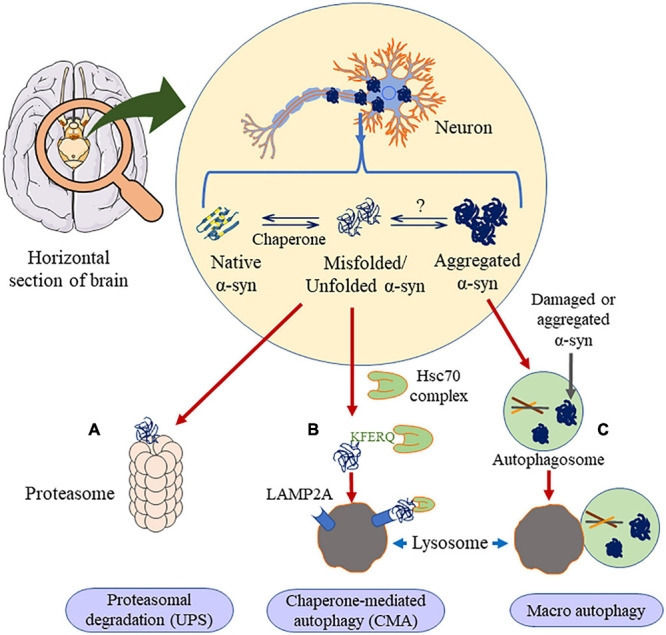
Clearance of α-syn occurs through three major ways. If the α-syn is in the unfolded dimer or the small oligomer state it undergoes proteasomal degradation **(A)** or chaperone mediated autophagy **(B)**, in presence of HSc70, α-syn is engulfed by lysosome through LAMP2A receptor. Else, if the aggregation is higher than oligomer state, it prefers macro autophagy **(C)**, where it forms autophagosome, which is engulfed by the lysosome. This figure was drawn using Motifolio.

In the case of removal of high molecular weight proteins, including oligomers and aggregates, the disposal mechanism shifts to autophagy (Lee et al., 2004). Based on its cargo delivery process, it is divided into CMA, macroautophagy and microautophagy. As of today, and to the best of our knowledge, there is no report on microautophagy clearing α-syn aggregation. The other two types of autophagic processes are discussed below (Figure 6).

Wild-type soluble α-syn is efficiently degraded in lysosomes by CMA, but the mutant α-syn is poorly degraded by CMA despite having an affinity for the CMA receptor. The lysosome-associated Hsc70 (lHsc70) protein helps in translocation of the targeted substrates for degradation (Chiang et al., 1989; Agarraberes et al., 1997). Mutant α-syn (A53T and A30P) also inhibits CMA substrates and lysosomal uptake that results in compensatory activation of macroautophagy (Cuervo et al., 2004). α-syn monomers and dimers, but not oligomers, are degraded via CMA (Martinez-Vicente et al., 2008; Xilouri et al., 2009). Alvarez-Erviti et al. (2010) had reported that decreased expression of LAMP2A, slows down the degradation of wild-type α-syn (Alvarez-Erviti et al., 2010). Hence, it was concluded that CMA is not involved in the degradation of misfolded α-syn directly. In contrast, Wu J.-Z. et al. (2019) reported that two bioactive ingredients dihydromyricetin and salvianolic acid B extracted from natural medicinal plants downregulate α-syn aggregation by activating both CMA and macroautophagy processes (Wu J.-Z. et al., 2019).

Endoplasmic reticulum stress is mainly an outcome of accumulated misfolded proteins, for example, α-syn, that undergoes ER associated degradation (ERAD) (McCracken and Brodsky, 2003). Misfolded/mutated proteins impair the ERAD system and contribute to PD pathogenesis (Lehtonen et al., 2019). GA regulates post-translational protein modifications, for example, glycosylation and proteolytic cleavage that occurs in the ER (Rabouille and Haase, 2016). Misfolded α-syn inhibits ER-Golgi transportation and leads to the aggregation of proteins in ER and triggers cell death in PD (Cooper et al., 2006; Wang and Hay, 2015). Furthermore, α-syn inhibits Rab1a which not only alters the ER-Golgi transportation, but also causes mislocalization of Atg9 trafficking, thereby, inhibiting autophagy (Winslow et al., 2010; Xilouri et al., 2016; Tomás et al., 2020).

Inositol-requiring enzyme 1 (IRE1), a key UPR signal activator, under ER stress clears protein aggregation via autophagy IRE1-X-box–binding protein 1 (XBP1) (Sado et al., 2009, 1; Fouillet et al., 2012; Valdés et al., 2014, 1; Ghavidel et al., 2015; Adams et al., 2019). On the other hand, Yan et al. (2019) reported that α-syn accumulation promotes neuronal death in *Drosophila* model of PD through the hyperactivation of IRE1 via the c-Jun N-terminal kinase (JNK)-dependent manner (Yan et al., 2019). Further research on the exact mechanisms of α-syn clearance will help understand the neuroprotective role of IRE1. Mesencephalic astrocyte-derived neurotrophic factor (MANF), also known as ARMET (arginine-rich mutated in early-stage tumors) is present in ER and promotes neuronal cell survival through UPR regulation (Apostolou et al., 2008; Renko et al., 2018; Wang et al., 2021). ER stress triggers the accumulation of misfolded α-syn (Colla et al., 2012a). MANF is shown to have neuroprotective activity in *in vitro* and *in vivo* models of PD (Voutilainen et al., 2009; Liu et al., 2018b). MANF is also reported to facilitate the cellular clearance of misfolded α-syn in a *Caenorhabditis elegans* model of PD. Inhibition of autophagy related genes by RNAi approach has been shown to decrease the expression of MANF suggesting its potential therapeutic role in PD (Zhang et al., 2018). In a recent clinical study, MANF level was also reported to be higher in the blood of PD patients. However further studies are required to reveal if MANF is a clinical marker for PD (Galli et al., 2019).

Zinc finger with KRAB and SCAN domains 3 (ZKSCAN3), a zinc-finger family DNA-binding protein initiates autophagosome biogenesis. However, it works antiparallel to TFEB (Chauhan et al., 2013). A30P mutant α-syn inhibits ZKSCAN3 and impairs autophagy in dopaminergic neuron (Lei et al., 2019).

Recent evidence proposes that AMPK signaling plays a crucial role in neurodegeneration. Rapamycin-induced initiation of autophagy, or AMPK agonists, promote the clearance of fibril-mediated α-syn pathology (Gao et al., 2019). Overexpressed AMPKα1 or α2 subunits integrate into the AMPK complex and protect dopamine neurons against human α-syn accumulation toxic effects (Bobela et al., 2017). AMPK also regulates PGC-1α, which is a transcriptional co-activator and master regulator of mitochondrial biogenesis (Wan et al., 2014). α-syn binds to the promoter sequence of PGC-1α and causes promoter methylation, a sporadic PD associated phenomenon which ultimately decreases PGC-1α expression (Su et al., 2015). AMPK inhibits mTORC1 by phosphorylating Raptor (Gwinn et al., 2008), along with indirect phosphorylation and activation of TSC2 (Inoki et al., 2003). unc-51-like autophagy activating kinase 1 (ULK1) drives autophagosome formation whilst mTORC1 suppresses (under nutrient condition) autophagy by phosphorylating ULK1 at Ser 757. In contrast, phosphorylation of ULK1 at Ser 317, Ser 777 or Ser 555 by AMPK promotes autophagy (Egan et al., 2011; Kim et al., 2011). Various reports suggest on the protective role of AMPK against the toxicity of both intracellular and extracellular α-syn (Choi et al., 2010; Wu et al., 2011; Dulovic et al., 2014; Anandhan et al., 2017; Bobela et al., 2017; Gao et al., 2019). α-syn is suggested to reduce AMPK phosphorylation and downstream target Raptor in SH-SY5Y neuroblastoma cells (Dulovic et al., 2014). However, several conflicting reports on AMPK signaling data are available. For example, Kim et al. (2013) and Xu et al. (2014) reported that activation of AMPK and inactivation of Akt causes neuronal cell death via inhibition of the mTOR pathway (Kim et al., 2013; Xu et al., 2014). Along similar lines, AMPK activation is also reported to trigger the aggregation of α-syn in primary neurons (Jiang et al., 2013). Future molecular investigations will help understand the role of AMPK in PD.

Cellular clearance of expired or damaged organelles is processed by autophagy, including the selective autophagy processes such as, (mitochondria) mitophagy, (peroxisomes) pexophagy, (ribosomes) ribophagy and parts of the nucleus involved in nucleophagy (Martinez-Vicente and Cuervo, 2007; Senkevich and Gan-Or, 2019). α-syn has been reported to impair mitophagy in PD (Shaltouki et al., 2018). α-syn exosome/extracellular vesicle (EV) fractions range from 60 to 160 nm in diameter, and are cleared by ALP. Inhibition of ALP increases α-syn levels (Alvarez-Erviti et al., 2011; Danzer et al., 2012; Minakaki et al., 2018). α-syn fibrils are transported to an endosomal compartment and lysosomes. The lysosomal inhibition is shown to accumulate α-syn aggregations, supporting the autophagy/lysosomal clearance pathway.

## Strategies That Facilitate Neuronal Clearance of α-Synuclein

### Reducing α-Synuclein Production

**FIGURE 7:**
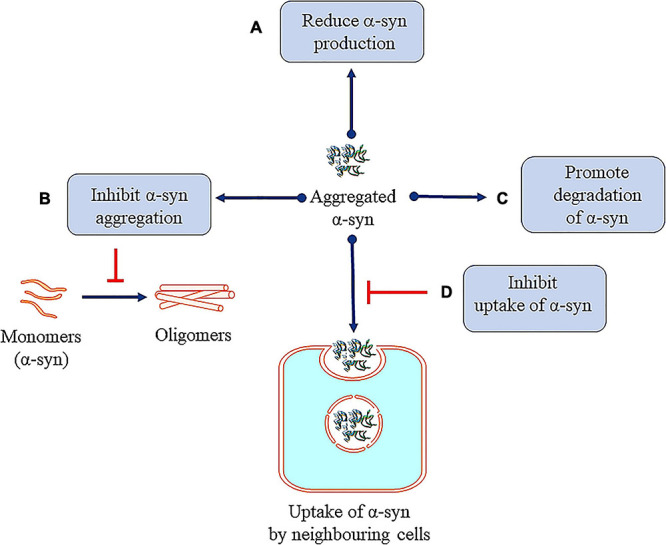
Therapeutic approaches against toxic α-syn. **(A)** Reducing α-syn production. **(B)** Inhibiting α-syn aggregation. **(C)** Promoting degradation of α-syn. **(D)** Inhibition of uptake of α-syn by neighboring cells.

Many research efforts are focused on protecting neuronal cells from α-syn toxicity, for example, reducing the synthesis of α-syn (Figure 7) by the infusion of siRNA in the hippocampal and cortical regions of mice (Lewis et al., 2008). In another study, injecting siRNA-containing exosomes is shown to lead to decrease in α-syn in the SNpc of Ser 129D α-syn transgenic mice (Cooper et al., 2014). α-syn propagation is also shown to participate in the neurotoxicity process and here the C-terminus (CT) of the protein plays a significant role (Games et al., 2014). Hence, monoclonal antibodies 1H7, 5C1, or 5D12 that target the CT, decrease α-syn in neurons and rescue TH in striatum which have been reported to improve motor ability and memory deficits (Games et al., 2014). Selective silencing of mutant SNCA gene has been shown to reverse the pathogenic characteristics of mutated α-syn while preserving the physiological functions of the native α-syn. These effects are consistently observed both in *in vitro* and *in vivo* studies using lentivirus mediated RNA interference (Sapru et al., 2006; Takahashi et al., 2015). Naked small interfering RNA (siRNA) is shown to reduce endogenous SNCA in hippocampus region, *in vitro* and *in vivo* models of PD. This has been translated to potential neuroprotective effect in α-synucleinopathies (Lewis et al., 2008). Also, Zharikov et al. (2015) have reported that knockdown of α-syn exerts neuroprotective role in a rotenone model of PD (Zharikov et al., 2015). Also, AAV vectors expressing miSyn4 siRNAs are reported to downregulate the α-syn (overexpressed) in mice (Kim et al., 2017). However, long-term RNAi knockdown of α-syn did not show any beneficial effects on dopaminergic functions in the adult rats (Zharikov et al., 2019).

### Inhibiting α-Synuclein Aggregation

Some reports have focused on inhibiting the aggregation of α-syn. Bae et al. (2012) used antibodies targeting the Fcγ receptors present on the surface of microglia to inhibit microglial triggered α-syn aggregation. Fonseca-Ornelas et al. (2014) reported that porphyrin phtalocyanine tetrasulfonate delays the aggregation of vesicle bound α-syn in H4 neuroglioma cells. Similarly, an α-syn protofibril-selective monoclonal antibody (mAb47) is shown to decrease its aggregation in A30P α-syn mutant mouse model (Lindström et al., 2014). Novel compounds NPT200-11 (Price et al., 2018) and NPT100-18A (Wrasidlo et al., 2016) are also reported to inhibit the aggregation of α-syn in preclinical models. It must be mentioned that compound NPT200-11 has cleared phase 1 of clinical trials (Table 2). A novel compound PBT434, is reported to slow down the progression of PD in hA53T α-syn transgenic mouse (Finkelstein et al., 2017). First generation epitope vaccines targeting the aggregated α -syn, are reported to be immunogenic in B6SJL mice (Ghochikyan et al., 2014). Heat shock proteins (HSP), especially small HSPs are molecular chaperones which have also been reported to inhibit α-syn aggregation in both *in vitro* and *in vivo* (McLean et al., 2002; Klucken et al., 2004; Gorenberg and Chandra, 2017).

**TABLE 2:** List the clinical trials targeting α-syn.

**Purpose/aim of the study**	**Drugs**	**Phase**	**Status**	**NCT number**
To find the presence of pathological α-syn within the CSF	–	–	Not yet recruiting	NCT04266457
To measure α-syn in peripheral body tissues and fluids in Parkinson’s disease	DaTSCAN (ioflupane-123I)		Completed	NCT02572713
To compare oligomeric and phosphorylated α-syn assay in cerebrospinal fluid and blood	–		Completed	NCT03170063
Nilotinib’s ability to alter the abnormal protein build up in Parkinson disease and diffuse Lewy body disease patients	Nilotinib	Phase 1	Completed	NCT02281474
EGCG as putative neuroprotective agent	Polyphenol (-)-epi-gallocatechin gallate (EGCG)	Phase 3	Completed	NCT02008721
Inhibition of the α-syn aggregations	NPT200-11	Phase 1	Completed	NCT02606682
Monoclonal antibody, single ascending dose	PRX002	Phase 1	Completed	NCT02095171
Monoclonal antibody, multiple ascending dose study	PRX002	Phase 1	Completed	NCT02157714
A study to evaluate the efficacy of Prasinezumab	PRX002	Phase 2	Active, not recruiting	NCT03100149
Vaccine (active immunotherapy) specific for α-syn	AFFITOPE^®^ PD01A	Phase 1	Completed	NCT01568099
Follow-up study to assess one boost immunization with AFFITOPE^®^ PD01A	AFFITOPE^®^ PD01A	Phase 1	Completed	NCT02216188
Extension study for patients with Parkinson’s disease after immunization with AFFITOPE^®^ PD01A	AFFITOPE^®^ PD01A	Phase 1b	Completed	NCT01885494
Tolerability and safety of AFFITOPE^®^ PD01A	AFFITOPE^®^ PD01A	Phase 1	Withdrawn	NCT02758730
Single-ascending dose study of BIIB054 in healthy participants and early PD	BIIB054	Phase 1	Completed	NCT02459886
Evaluating the efficacy, safety, pharmacokinetics, and pharmacodynamics of BIIB054 in PD patients	BIIB054	Phase 2		NCT03318523
Expression patterns of GALIG gene (α-synuclein interacts with Cytogaligin, a protein produced by the proapoptotic GALIG gene)	–	–	Completed	NCT02923297
Diagnostic and prognostic biomarkers in Parkinson disease (PROBE)	–	–	Active, not recruiting	NCT00653783
Phenylbutyrate response as a biomarker for α-syn clearance from the brain	Glycerol phenylbutyrate	Phase 1	Active, not recruiting	NCT02046434
A pilot biomarker study assessing α-syn aggregates across biofluid reservoirs in patients with synucleinopathies	–	–	Recruiting	NCT04020198
α-Syn as a marker for early diagnosis of Parkinson’s disease in skin biopsy.	–	–	Enrolling by invitation	NCT01380899
Inhibition of α-syn cell–cell transmission by NMDAR blocker	Memantine	Phase 3	Recruiting	NCT03858270
Alpha-synuclein level in saliva to differentiate between idiopathic Parkinson disease	–	–	Recruiting	NCT03156647
Diagnosis of Parkinson’s disease by means of submandibular gland needle biopsy	–	–	Recruiting	NCT04264273
Effects of lithium therapy on blood-based therapeutic targets in Parkinson’s disease.	Lithium	Phase 1	Recruiting	NCT04273932
Biomarker analysis for GBA associated Parkinson’s disease	–	–	Recruiting	NCT03811496
Oligomeric α-syn CSF levels	–	–	Recruiting	NCT02114242
Measuring serum alpha synuclein autoantibodies	–	–	Recruiting	NCT04062279
Idiopathic rapid eye movement (REM) sleep behavior disorder (RBD), for the purpose of preparing for a clinical trial of neuroprotective treatments against synucleinopathies.	–	–	Recruiting	NCT03623672
A first-in-human study of single and multiple doses of anle138b in healthy subjects	anle138b	Phase 1	Recruiting	NCT04208152
Drug against glucocerebrosidase (GBA) gene mutation in PD	GZ/SAR402671	Phase 2	Active, not recruiting	NCT02906020
Multiple ascending dose study of MEDI1341 in patients with PD	MEDI1341	Phase 1	Active, not recruiting	NCT04449484
Lu AF82422 in healthy non-Japanese and Japanese subjects and in patients with PD	Lu AF82422	Phase 1	Recruiting	NCT03611569

### Promoting Degradation of α-Synuclein

Increasing α-syn clearance through lysosomal/or autophagic process also leads to decrease in the cellular levels. Decressac et al. (2013) reported that stimulation of TFEB function or blocking of mTOR prevents the degeneration of dopaminergic neurons caused by α-syn toxicity (Decressac et al., 2013). Passive immunization with monoclonal α-syn antibodies (9E4) is also shown to clear α-syn aggregation via a lysosomal pathway (Masliah et al., 2011; Bae et al., 2012).

Additionally, deficiency of GD-linked glucocerebrosidase (GCase) is also reported to impair the lysosomal proteolytic enzyme in primary cultures or induce hiPSC neurons, triggering aggregation of α-syn (Mazzulli et al., 2011). Toward this end, increasing the GCase activity by AAV-GBA1 (gene encoding glucocerebrosidase) intra-cerebral gene delivery has also been shown to protect against α-syn toxicity in rodents (Rocha et al., 2015). Furthermore, NCGC607 (Aflaki et al., 2016) and NCGC00188758 (Mazzulli et al., 2016) (new leads against α-syn) are shown to improve the GCase activity and decrease α-syn accumulation in human neurons. Interestingly, Kalekrein 6 (KLK6) is a serine protease in PD whose expression levels are inversely correlated toα-syn and recombinant KLK6 is reported to degrade of extracellular α-syn directly (Pampalakis et al., 2016).

Several other studies of active and passive immunization against α-syn aggregation are reported and are shown to be neuroprotective (Masliah et al., 2005, 2011; Sanchez-Guajardo et al., 2013; Christiansen et al., 2016). Here, drugs like PRX002 (Table 2) (a humanized IgG1 monoclonal antibody), that has successfully entered in Phase 2 (Jankovic et al., 2018) clinical trials (NCT02157714) needs a special mention. Further, safety and tolerability is being tested in the PD patients (Schenk et al., 2017).

### Inhibition of Uptake of α-Synuclein by Neighboring Cells

α-Synuclein monoclonal antibodies (mAbs) are reported to inhibit propagation and uptake of α-syn and prevent the aggregation of α-syn in a mouse model (Tran et al., 2014). Mao et al. (2016) have demonstrated that α-syn fibrils bind to lymphocyte-activation-gene 3 (LAG3) protein and initiate endocytosis into neuronal cells (Mao et al., 2016). The involvement of other proteins in the initiating endocytosis is suggested (Shrivastava et al., 2015). Further research in this area is ongoing and will help in understanding the role of endocytosis in removal of pathogenic α-syn. In line with this, it is important to mention that Gustafsson et al. (2017) had also reported that, inhibiting Fcγ receptors (FcγRI and FcγRIIB/C) results in reduced uptake of α-synu oligomer/protofibril (Gustafsson et al., 2017). Here, astrocytes are reported to take up α-syn preformed fibrils (pffs) via endocytosis process. Clusterin interacts with α-syn pffs in the extracellular compartment and the clusterin/α-syn complexes are internalized by astrocytes. To this end, clusterin knock-out primary astrocytes and clusterin knock-down hiPSC-derived astrocytes are also reported that limits the uptake of α-syn pffs by the cells (Filippini et al., 2021).

## “Janus-Faced” α-Synuclein

α-Synuclein and cysteine-string protein-alpha (CSPalpha) are present abundantly in SV. CSPalpha plays a vital role in neuronal growth and its deletion is shown to cause progressive neurodegeneration in mouse model. Interestingly, abnormal expression of α-syn causes neurodegeneration and motor impairment due to the deletion of CSPalpha. Also, α-syn is shown to inverse the soluble *N*-ethylmaleimide-sensitive factor attachment protein receptor (SNARE)-complex assembly, a pathological impediment observed in the CSPα knockout mice (Chandra et al., 2005). α-syn binds at the N-terminus of SNARE protein synaptobrevin-2 by its C-terminus (Burré et al., 2010). Greten-Harrison et al. (2010) demonstrated that deletion of α-syn causes alterations in the synaptic structure and leads to transferable and age dependent neuronal dysfunction. It further causes decrease in synapse size by ∼30% both *in vivo* and *in vitro* (Greten-Harrison et al., 2010). These data indicate the neuroprotective roles of α-syn at the synapse. However, Darios et al. (2010) reported indirect inhibitory effect of α-syn on SNARE-complex assembly by inhibition of arachidonic acid. Arachidonic acid is reported to stimulate SNARE-complex formation and exocytosis (Darios et al., 2010). It is widely known that the mutated form of α-syn is linked to PD pathology (Chandra et al., 2005; Stefanis, 2012).

Furthermore, α-syn is also reported to exert protection of neurons against various apoptotic stimuli (da Costa et al., 2000). Additionally, the involvement of α-syn in various biological functions such as synaptic transmission, calcium regulation, mitochondrial homeostasis, gene expression, protein phosphorylation cannot be ignored (Sharon et al., 2001; Ellis et al., 2005). α-syn interacts in SV and SNARE proteins, mediating the vesicular transport to presynaptic membrane (Maroteaux et al., 1988; Burré et al., 2010, 2012). α-syn inhibits TH (Perez et al., 2002; Perez and Hastings, 2004) and its phosphorylation either by increasing PP2A activity or by altering the binding sites of TH for phosphorylation (Peng et al., 2005; Wang et al., 2009). Supporting the phenomenon of DA release from presynaptic membrane of neuronal cells, α-syn also inhibits AADC, an inhibitor of DA synthesis (Tehranian et al., 2006). Moreover, α-syn is shown to interact with the DAT, modulate DAT activity (increasing or decreasing) and increase the amount of VMAT on vesicles (Lee et al., 2001; Dauer et al., 2002; Wersinger and Sidhu, 2003; Wersinger et al., 2003; Fountaine and Wade-Martins, 2007; Fountaine et al., 2008).

The *SNCA* expression in terms of PD pathogenesis is delicately balanced. Supporting this concept, a clinical study on well characterized PD patients described that the low repeat REP1 allele, a complex microsatellite (259 base pairs; resulting in decreased *SNCA* expression) is associated with motor and cognitive dysfunctions, whereas the high-repeat REP1 allele (263 base pairs; increases *SNCA* expression) is associated with improving the motor and non-motor symptoms like cognition (Markopoulou et al., 2014). Contradictory data by Corrado et al. (2018) reported that, REP1 allele (263 base pairs) is associated with inferior cognitive outcome (Corrado et al., 2018) in PD. In a clinical patients based study it was reported that, long REP1 alleles are associated with motor and non-motor functions in PD (Ng et al., 2019). Adding to this study, the role of *SNCA* Rep1 allele length in non-motor functions as well as depression in the early PD patients was also reported (Yong et al., 2020). It was argued that Markopoulou et al. (2014) collected the data through telephonic interviews and hence there could be possibility of miscommunication. Secondly, the biological effect of REP1 allele could also vary with the patients, especially in different ethnic groups. Hence, it was concluded that future studies with more patients are required to resolve these contradictory findings.

Interestingly, in another report both aggregation and knockdown of α-syn were reported to impair mitochondrial Ca^2+^ homeostasis and induce toxicity (Calì et al., 2012). Ludtmann et al. (2016) had also reported that monomeric α-syn enters the mitochondria and enhances ATP synthase function (Ludtmann et al., 2016). α-syn was also reported to participate in the physiological functions of mitochondria like fusion, ETC, and VDAC permeability (Ellis et al., 2005; Kamp et al., 2010; Rostovtseva et al., 2015). Supporting the protective role of α-syn, Seo et al. (2002) reported that at nanomolecular concentrations, α-syn is shown to protect the primary neurons against oxidative stress (Seo et al., 2002). Recently, Carmo-Gonçalves et al. (2020) had studied the role of monomeric and fibrillar α-syn on mesencephalic dopaminergic neurons in primary cultures using neurotoxic salsolinol and 3,4-dihydroxyphenylacetaldehyde (DOPAL). They reported that the protective properties of monomeric α-syn involve the inhibition of caspase 3 mediated apoptosis (Carmo-Gonçalves et al., 2020).

Furthermore, increased oxidative stress is shown to trigger the aggregation of α-syn in PD (Scudamore and Ciossek, 2018). Accumulations and overexpression of α-syn further triggers α-syn misfolding (Hsu et al., 2000; Lee et al., 2002b; Chinta et al., 2010) leading to mitochondrial fragmentation and dopaminergic cell death (Menges et al., 2017). Here, it must be mentioned that overexpression and aggregation of α-syn is linked to decrease in the neurotransmitters and consequent motor dysfunctions (Larsen et al., 2006; Gaugler et al., 2012; Scott and Roy, 2012).

**FIGURE 8:**
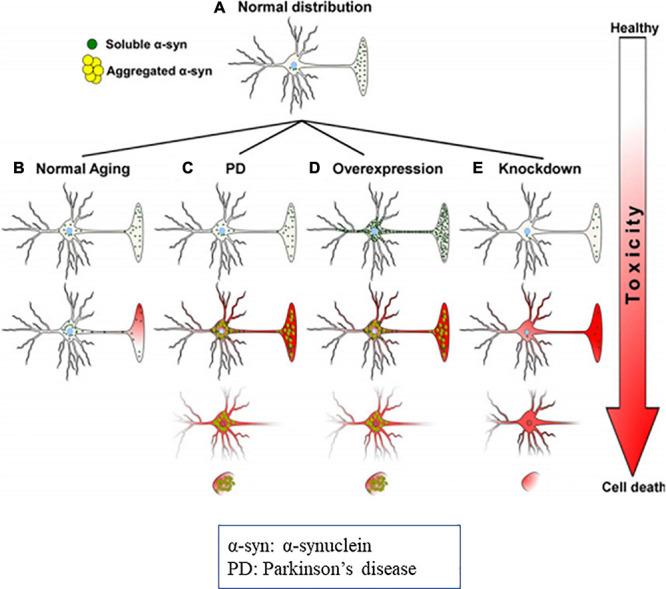
Diagrammatic representation of α-syn distribution and its associated toxicities (Cell death). **(A)** Physiological distribution of α-syn in healthy neurons mainly in presynaptic terminal. **(B)** During the aging distribution of α-syn spreads from the presynaptic terminal to the soma, that causes subsequent toxicity. In PD, due to genetic mutations, oxidative stress, α-syn aggregates and produce toxicities and cell death. **(C)** Overexpression or molecular crowding of α-syn causes toxicities. **(D)** Knockdown of α-syn below threshold (protein concentration) results in cell death. Image reused as per Creative Commons Attribution-Non-commercial-NoDerivs License (Benskey et al., 2016).

It was observed that, in α-synucleinopathy, some neurons express abundant Lewy pathology than other neuronal types. For example, dopaminergic, noradrenergic, cholinergic and the glutamatergic neurons express abundant α-syn aggregation, whereas, most of the GABAergic neurons are spared (Wakabayashi et al., 1995; Spillantini et al., 1998; Gómez-Tortosa et al., 2001; Del Tredici and Braak, 2013; Hall et al., 2014; Kay et al., 2015; Taguchi et al., 2019). Expression of α-syn protein is positively correlated with susceptibility to aggregate (Figure 8) (Wakabayashi et al., 1995; Erskine et al., 2018; Taguchi et al., 2019). Thus, conflicting reports on α-syn in literature need to be resolved.

## Conclusion

α-Synuclein is reported to be involved in the DA release in the synapse and also has a neuroprotective roles in apoptotic stimuli. There are many scientific reports which establish the physiological role of α-syn in healthy individuals. α-syn is a vital component of LBs which are the major pathological hallmarks in PD. Reports also suggest that misfolded α-syn can travel from cell-to-cell (Freundt et al., 2012; Domert et al., 2016; Tyson et al., 2017; George et al., 2019; Rey et al., 2019). The point mutations that result in change of amino acid in α-syn (A30P, E46K, H50Q, G51D, A53E, and A53T) are studied along with their aggregation kinetics in PD. Further studies confirming the pathogenic mutations and aggregation could help to target α-syn and understand its role in disease pathogenesis. Several studies are being conducted that target pathogenic α-syn and cause impairing of the autophagy and proteasomal processes. Furthermore, the pathogenic origin of α-syn is being explored in relation to gut dysbiosis. Early diagnosis of PD is a major field of interest in modern science. The identification of a biomarker which can detect α-syn toxicity could potentially lead to novel strategies for effective PD diagnosis and treatment. There is a need to collate and present latest data on α-syn and provide a unified view of the protein. This review is an attempt in this direction and aims to help understand the pathophysiological role of α-syn and its aggregation in PD.

## Author Contributions

BR contributed to the investigation, writing – original draft, writing – review and editing and visualization. AM and ST contributed to the writing – review and editing. AB contributed to the writing – review and editing, and visualization. AS, CP, AK, and SB contributed to the validation. MS contributed to formal analysis and writing. SC contributed to the conceptualization, methodology, validation, and formal analysis. All authors contributed to the article and approved the submitted version.

## Conflict of Interest

The authors declare that the research was conducted in the absence of any commercial or financial relationships that could be construed as a potential conflict of interest.

## Abbreviations

α -syn: α -synuclein
A β: amyloid- β
AMPK: AMP-activated protein kinase
AADC: aromatic amino acid decarboxylase
AFM: atomic force microscopy
CMA: chaperone-mediated autophagy
DMVN: dorsal motor nucleus of the vagus nerve
DAT: dopamine transporter
ER: endoplasmic reticulum
ETC: electron transport chain
ENS: enteric nervous system
ERAD: endoplasmic reticulum associated degradation
GI: gastrointestinal
GA: Golgi apparatus
hiPSC: human pluripotent stem cells
IPANs: intrinsic primary afferent neurons
IRE1: inositol-requiring enzyme 1
lHsc70: lysosome-associated Hsc70
LAMP2A: lysosome-associated membrane protein type 2a
LBs: Lewy bodies
LBD: Lewy body disorders
mTOR: mammalian target of rapamycin
NO: nitric oxide
NMR: nuclear magnetic resonance
OB: olfactory bulb
PP2A: protein phosphatase 2A
PD: Parkinson’s disease
RAb1A: Ras-related protein Rab-1A
SNpc: substantia nigra pars compacta
SV: synaptic vesicles
TFEB: transcription factor EB
TH: tyrosine hydroxylase
TGN: *trans*-Golgi network
UPR: unfolded protein response
ULK1: Unc-51-like autophagy activating kinase 1
VMAT: vesicular monoamine transporter
XBP: X-box –binding protein 1
ZKSCAN3: zinc finger with KRAB and SCAN domains 3.
